# The Role of Pulmonary Collectins, Surfactant Protein A (SP-A) and Surfactant Protein D (SP-D) in Cancer

**DOI:** 10.3390/cancers16183116

**Published:** 2024-09-10

**Authors:** Maciej Cedzyński, Anna S. Świerzko

**Affiliations:** Laboratory of Immunobiology of Infections, Institute of Medical Biology, Polish Academy of Sciences, Lodowa 106, 93-232 Łódź, Poland; aswierzko@cbm.pan.pl

**Keywords:** cancer, collectin, pulmonary surfactant, surfactant protein A (SP-A), surfactant protein D (SP-D)

## Abstract

**Simple Summary:**

Pulmonary surfactant prevents alveolar collapse by reducing surface tension at the air–liquid interface. It contains two hydrophilic lectins, called SP-A and SP-D. They are factors of innate immune defence but also contribute to the surfactant structure and homeostasis. SP-A and SP-D recognise pathogen- or danger-associated molecular patterns (PAMPs, DAMPs), which enables opsonisation or agglutination of non-self or altered/abnormal self cells and contributes to their clearance. The term “cancer” includes a variety of diseases, often incurable, difficult to diagnose and fatal. This short review summarises anti- and pro-tumorigenic associations of SP-A and SP-D as well as perspectives of their usefulness in cancer diagnosis and therapy.

**Abstract:**

Surfactant proteins A and D (SP-A and SP-D) belong to the collectin subfamily of C-type oligomeric lectins. They are pattern-recognition molecules (PRMs), able to recognise pathogen- or danger-associated molecular patterns (PAMPs, DAMPs) in the presence of Ca^2+^ cations. That property enables opsonisation or agglutination of non-self or altered/abnormal self cells and contributes to their clearance. Like other collectins, SP-A and SP-D are characterised by the presence of four distinct domains: a cysteine-rich domain (at the N-terminus), a collagen-like region, an α-helical neck domain and a globular carbohydrate-recognition domain (CRD) (at the C-terminus). Pulmonary surfactant is a lipoprotein complex, preventing alveolar collapse by reducing surface tension at the air–liquid interface. SP-A and SP-D, produced by type II alveolar epithelial cells and Clara cells, are not only pattern-recognition molecules but also contribute to the surfactant structure and homeostasis. Moreover, they are expressed in a variety of extrapulmonary sites where they are involved in local immunity. The term “cancer” includes a variety of diseases: tumours start from uncontrolled growth of abnormal cells in any tissue which may further spread to other sites of the body. Many cancers are incurable, difficult to diagnose and often fatal. This short review summarises anti- and pro-tumorigenic associations of SP-A and SP-D as well as perspectives of their usefulness in cancer diagnosis and therapy.

## 1. The Collectin Subfamily

**Figure 1:**
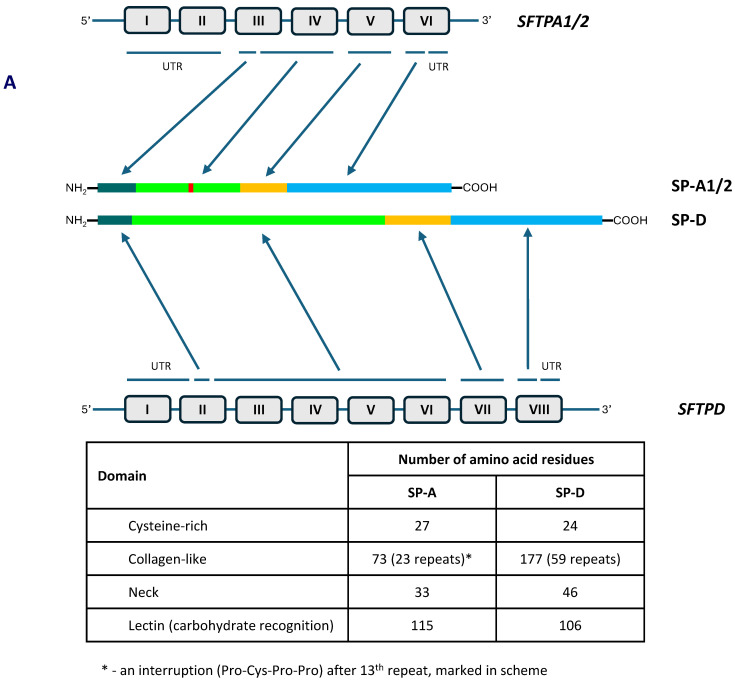

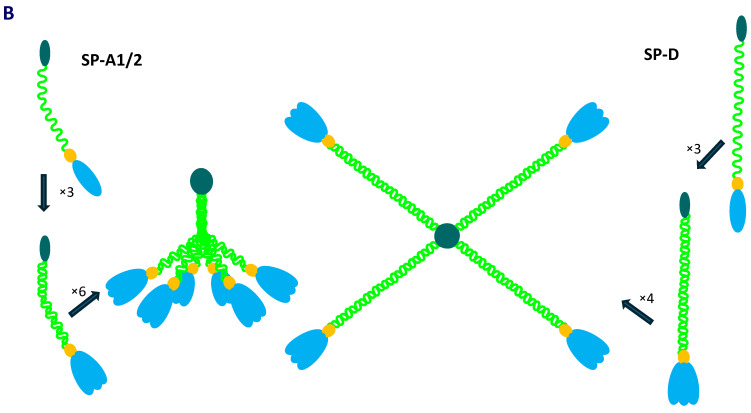
Organisation of *SFTPA1/2* and *SFTPD* genes and domain organisation of polypeptide chains of SP-A and SP-D (**A**). Schematic structure of single chains, basic triplet subunits and oligomers of SP-A and SP-D (**B**).

The human collectin subfamily of C-type lectins includes six oligomeric pattern-recognition molecules (PRM): mannose-binding lectin (MBL) (also called mannan-binding lectin), collectin-10 (CL-10) (or collectin-liver 1, CL-L1), collectin-11 (CL-11) (or collectin-kidney 1, CL-K1), collectin-12 (CL-12) (or collectin-placenta 1, CL-P1), surfactant protein A (SP-A) and surfactant protein D (SP-D) [1,2]. They recognise pathogen- or danger-associated molecular patterns (PAMPs, DAMPs) in the presence of Ca^2+^ cations, enabling opsonisation or agglutination of non-self or altered/abnormal self cells and contributing to their clearance. Moreover, MBL, CL-10, CL-11 and CL-12 are able to activate the complement system, extending the range of their participation in the immune response, while SP-A and SP-D lack this property [1,2]. In general, the basic subunit of collectin molecules consists of three, usually identical, chains. However, heterotrimers built up from CL-10 and CL-11 (their higher oligomer is called CL-LK) or two different SP-A polypeptides (discussed below) are not uncommon. Those subunits undergo further oligomerisation, forming dimers, trimers, tetramers or hexamers of basic triplets. That enables strengthening of ligand binding capacity, which is relatively low for a single CRD [1,2]. Collectins are characterised by the presence of four distinct domains: a cysteine-rich domain (at the N-terminus), a collagen-like region, an α-helical neck domain and a globular carbohydrate-recognition domain (CRD) (at the C-terminus). The collagenous domain includes Gly-X-Y repeats (where X and Y correspond to any amino acid residues) [1,2]. Figure 1 demonstrates structure of SP-A and SP-D as well as organisation of corresponding genes.

## 2. Pulmonary Surfactant Collectins: SP-A and SP-D

Pulmonary surfactant is a lipoprotein complex, preventing alveolar collapse by reducing surface tension at the air–liquid interface. Proteins constitute approximately 10% of the complex. Two hydrophobic proteins, SP-B and SP-C, are involved in controlling surface tension and stabilisation of the surfactant. Furthermore, the latter is considered to influence the innate immune response, due to its ability to bind bacterial lipopolysaccharide (LPS, endotoxin). Hydrophilic collectins, SP-A and SP-D, produced by type II alveolar epithelial cells and Clara cells, are not only pattern-recognition molecules but also contribute to the surfactant structure and homeostasis [3]. Two other surfactant proteins, SP-G (or surfactant-associated protein 2, SFTA2) and SP-H (or SFTA3), although structurally different, have physicochemical properties resembling SP-B and SP-C [4].

**Figure 2:**
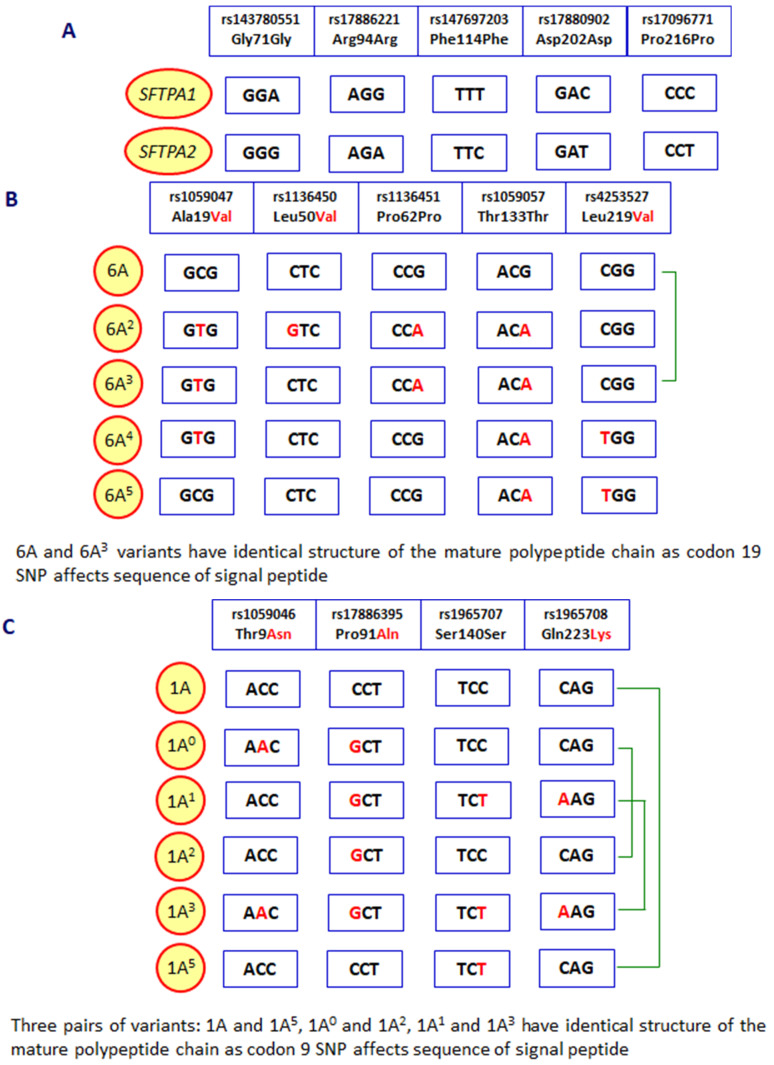
Polymorphic sites distinguishing between the *SFTPA1* and *SFTPA2* genes (**A**), variants of the *SFTPA1* gene (**B**) and the *SFTPA2* gene (**C**). Green braces mark *SFTPA1* or *SFTPA2* variants corresponding to mature SP-A1 or SP-A2 proteins with identical sequences.

As mentioned, human pulmonary surfactant protein A has two distinct forms, called SP-A1 and SP-A2, which may form homo- or heterotrimeric subunits. Despite high similarity, they are encoded by separate genes (*SFTPA1* and *SFTPA2*), each encompassing six exons, being in linkage disequilibrium. They are localised to chromosome 10 (10q21-24), in opposite transcriptional orientation. Both *SFTPA1* and *SFTPA2* are highly polymorphic. Their variants, called 6A, 6A^2^, 6A^3^, 6A^4^ and 6A^5^ and 1A, 1A^0^, 1A^1^, 1A^2^, 1A^3^ and 1A^5^, respectively, corresponding to fourteen single-nucleotide polymorphisms (SNPs), have been established (for details, see Figure 2) and are commonly used in analyses concerning SP-A clinical associations [2,3,5]. Since two SNPs (within codons 9 and 19) affect sequences of signal peptides but not structures of mature protein (Figure 2), expression of certain pairs of genetic variants [6A–6A^3^ (*SFTPA1*); 1A–1A^5^, 1A^0^–1A^2^ and 1A^1^–1A^3^ (*SFTPA2*)] results in synthesis of identical products [6]. The 35 kDa polypeptide chains of both SP-A1 and SP-A2 consist of 248 AA (Figure 1). That collectin recognises PAMPs/DAMPs containing residues of such monosaccharides as N-acetyl-D-mannosamine (D-ManNAc), D-mannose (D-Man), L-fucose (L-Fuc), D-maltose (D-Mal), D-glucose (D-Glc) or N-acetyl-D-glucosamine (D-Glc-NAc) and other compounds, like galactosyl-ceramide, lactosyl-ceramide and nucleic acids [1,2,4,6,7,8].

The *SFTPD* (SP-D) gene, encompassing eight exons, is also localised to chromosome 10 (10q21-24). Like those encoding SP-A, it is highly polymorphic. Some *SFTPD* SNPs located in exons are related to amino acid exchanges [rs721917 (+32 T>C, M11T), rs2243639 (+478 A>G, T160A), rs3088308 (+868 T>A, S270T)]; several others are associated with modifications of the nucleotide sequence only [rs6413520 (+75 T>C, S25S), rs1051246 (+858 T>C, A286A)] [3,9,10]. Those mentioned *SFTPD* polymorphisms (and some others) are considered clinically relevant. This review is however limited to their associations with cancer, discussed below.

The SP-D polypeptide chain (43 kDa) is composed of 355 amino acid residues (Figure 1). Basic trimers oligomerise into dodecamers (tetramers of subunits) which may further multimerise (up to 96 single chains). The carbohydrate-recognition domain of this collectin has affinity for the majority of sugars being recognised by SP-A (D-Mal, D-Man, L-Fuc, D-GlcNAc and D-Glc) but also recognises D-galactose (D-Gal), lactose (disaccharide), phosphatidylinositol, glucosyl-ceramide, DNA and RNA [2,4,6,8,11,12].

**Figure 3:**
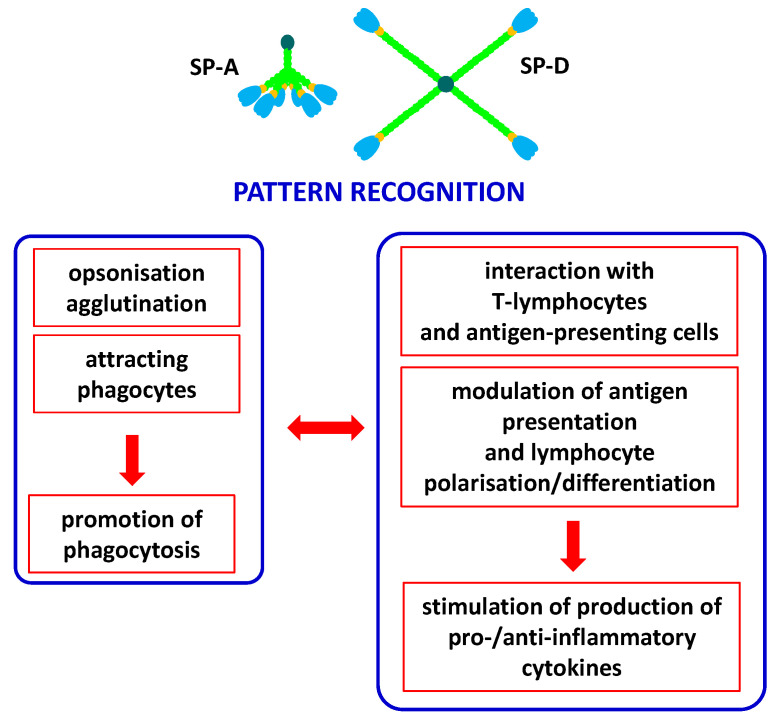
Major activities of SP-A and/or SP-D as players of the immune system. Pattern recognition (non-self or abnormal self, including pathogens, allergens and apoptotic cells) leads to variety of consequences, resulting in promotion of phagocytosis (**left box**) and promotion of cytokine production (**right box**). Both pathways of response are cross-talking and mutually dependent.

Pulmonary surfactant collectins are important components of the innate immune system, crucial for protection of the respiratory system from pathogens or allergens. Moreover, they are also expressed in a variety of extrapulmonary sites where they are involved in local immunity. Both SP-A and SP-D have been detected (at various levels) in the brain, gastrointestinal system, skin, salivary glands, eyes and lacrimal system, Eustachian tube, thymus, kidneys, ureter, bladder, testes, prostate, mammary glands, uterus, vagina, amniotic epithelium and placenta. Furthermore, SP-D can be found in the heart, trachea, ovaries, fallopian tubes and blood [4,13,14]. SP-A and SP-D act as chemoattractants for macrophages, promote phagocytosis, stimulate the production of pro- and anti-inflammatory cytokines and interact with antigen-presenting cells and T-lymphocytes. Importantly, despite clearance of pathogens/allergens, pulmonary collectins contribute to the removal of apoptotic cells via promoting their phagocytosis and then preventing adverse effects of their accumulation [3,14]. The involvement of pulmonary surfactant collectins in immune response is summarised in Figure 3.

## 3. The Role of SP-A and SP-D in Cancer

### 3.1. Lung Cancers

Most reports concerning associations of pulmonary surfactant collectins with malignancies relate to lung cancer. Lung cancer is the most frequently diagnosed cancer and the leading cause of cancer-associated mortality (causing approx. as many deaths as colorectal, breast and prostate cancers put together) [15,16,17,18,19]. In 2022, nearly 2.5 million new cases were diagnosed globally (12.4% of all cancers), and 1.8 million deaths were noted (18.7%) [19]. This high mortality is a reflection of difficulty of early diagnosis, high metastatic potential and often poor response to therapy [20,21]. Most tumours are classified as non-small-cell lung cancer (NSCLC, >80% cases) and include adenocarcinomas, squamous-cell carcinomas and large-cell carcinomas. The remaining <20% are small-cell lung carcinomas [17].

Kaczmarek et al. [22] found higher SP-A concentrations in malignant (from patients diagnosed with non-small-cell lung cancer) than non-malignant pleural effusions. Levels of this collectin in malignant samples correlated positively with stem cell factor (SCF) but inversely with macrophage colony-stimulating factor (M-CSF). High SP-A levels were accompanied by an increase in the number of M2 macrophages and a decrease in the number of M1 macrophages [22]. Earlier, high SP-A concentrations were observed in pleural effusions from approx. 40% of patients suffering from pulmonary adenocarcinoma but not from those diagnosed with other adenocarcinomas, other lung cancers or tuberculosis [23]. The same group suggested high SP-A and carcinoembryonic antigen (CEA) concentrations in pleural effusions to distinguish between lung adenocarcinoma and mesothelioma [24]. Takezawa et al. [25], with the use of immunostaining or RT-PCR, provided evidence of the expression of SP-A in cells present in pleural effusions from the majority of patients suffering from primary lung adenocarcinoma. Using immunohistochemistry, this collectin was also found in primary pulmonary adenocarcinoma cells inside tumours [17,26,27,28,29,30,31,32,33,34] and, like in pleural effusions, is proposed to be useful for discrimination of that disease from mesothelioma [29]. A high ratio of MUC1 mucin/SP-A expression appeared to predict a fatal outcome in patients diagnosed with small-size tumours [31]. Liu et al. [34] found no association of SP-A status with patient’s age, tumour differentiation or disease stage, but positive immunostaining was more frequent among patients with confirmed mutations of the *EGFR* (*HER1*) gene (characteristic for some cancers, including pulmonary adenocarcinomas, especially in female non-smoking Eurasian patients), encoding for the epidermal growth factor receptor [34]. Linnoila et al. [26] found the highest frequency (50%) of positive staining in adenocarcinomas of papillolepidic growth patterns and lower frequencies in other adenocarcinomas and other non-small-cell lung cancers. Although Suzuki et al. [32] observed no SP-A staining in other primary lung carcinomas or adenocarcinomas of other organs, they suggested napsin A (an aspartic protease, involved in pro-SP-B processing) to be a better disease marker. Furthermore, Zamecnik and Kodet [30] found small-cell lung carcinomas, squamous-cell carcinomas and carcinoid tumours SP-A to be negative, in contrast to 46% of adenocarcinomas and 25% of non-neuroendocrine large cell carcinomas, and suggested that SP-A immunostaining does not improve the diagnostic usefulness of thyroid transcription factor-1 (TTF-1). SP-D was in turn suggested to be useful as a marker of risk of early lung cancer in smokers and ex-smokers: its low concentration in bronchoalveolar lavage fluid (BALF) was found to be associated with progression of bronchial dysplasia [35]. Later, Yamaguchi et al. [36] found a significant relationship between high SP-D concentration in serum before therapy and longer progression-free survival in patients suffering from advanced non-small-cell lung carcinomas, treated with gefitinib. No such association was observed for SP-A [36]. Moreover, a high SP-D level was reported to be associated with a lower number of distinct metastases and to predict longer progression-free survival and overall survival in patients with lung adenocarcinoma with *EGFR* mutations, treated with tyrosine kinase inhibitor (TKI) [37], as well as overall survival in patients with NSCL undergoing stereotactic body radiotherapy [38]. On the other hand, Takahashi et al. [39] found higher concentrations of both SP-A and SP-D in sera from patients suffering from lung cancers who developed radiation pneumonitis (RP), in comparison with those without RP. Similarly, higher median SP-D was observed by Shiels et al. [40] in patients of various ethnicities (mainly Caucasian) compared with controls. High (fourth quartile) levels of this collectin were found to be a risk factor for developing disease [40]. Interestingly, SP-D and SP-A are able to supress EGFR-mediated signalling via different mechanisms. SP-D binds the EGFR due to recognition of oligomannose-type N-glycans by the lectin domain (CRD) (inhibitable by EDTA), while SP-A interacts with that receptor electrostatically [41,42].

Expression of SP-A at the mRNA level in primary lung adenocarcinoma cells was reported by Broers et al. [43] and Takahashi et al. [44]. Furthermore, SP-A- (as well as SP-C)-specific mRNA was also detected in the peripheral blood of one-third of primary NSCLC patients. It was suggested that their presence indicated a high risk of metastasis. Indeed, Betz et al. [45] observed SP-A and SP-C mRNA expression in metastatic pulmonary adenocarcinomas but not metastatic NSLC or extrapulmonary adenocarcinomas. No SP-A expression was found among cases of small-cell lung cancer, secondary lung tumours or non-malignant respiratory diseases [20].

Wang et al. [46] claimed two *SFTPA2* gene polymorphisms, T593C (F198S) and G692T (G231V), were associated with lung adenocarcinoma. The corresponding variant alleles lead to a change of structure in the SP-A2 lectin domain and therefore may affect its ability to recognise molecular patterns. Although SP-A was detectable in tumour cells from heterozygous patients, transfection of A549 cells with any of the mutated *SFTPA2* variants revealed poor protein expression, in contrast to the cells transfected with wild-type gene. It was speculated that impaired SP-A2 synthesis may lead to an imbalance of immunoregulation and result in pulmonary fibrosis or cancer [46]. The significance of SP-A polymorphisms affecting CRD structure/function was further explored in studies of familial interstitial lung diseases and cancer. The G532A (V178M), T631C (W211R) and C655T (R219W) single-nucleotide (amino acid) exchanges in the *SFTPA1* gene and T697A (W233R), G699C (W233C) and G713C (C238S) in the *SFTPA2* gene were associated with lung tumours in families of various ethnicities [47,48]. For the *SFTPD* gene, variant alleles for T32C (M11T) and intronic rs2245121 (G>A) were reported to be risk factors in Japanese and Chinese (smokers) populations [49,50]. Moreover, Grageda et al. [51] found lower *SFTPA2* mRNA/protein expression in pulmonary squamous-cell carcinoma and adenocarcinoma, accompanied by higher DNA methylation of the gene promoter in the former. Earlier, an association of hypermethylation of the *SFTPA1* (at SP-A1_370 and SP-A1_1080 CpG sites) and *SFTPD* (SP-D_1170 and SP-D_1370) with cancers of the same types was reported by Lin et al. [52]. Jiang et al. [53,54], investigating genomic profiles of patients diagnosed with stage I primary NSCLC, found some aberrations (including SP-A deletion), possibly associated with tumorigenesis and potentially useful as disease markers. Using fluorescence in situ hybridisation (FISH), they found ≤3% cells presenting loss of SP-A signals in normal lung tissues. In 25/28 tumour specimens, the lack of corresponding signal was observed in >6% cells. Importantly, that occurred significantly more often in samples from smokers compared with never-smokers. Moreover, SP-A deletions (and especially their high rate) predicted shorter survival time. It was also suggested that the SP-A copy number may be helpful in predicting appropriateness and effectiveness of adjuvant therapy [54]. Interestingly, Linnoila et al. [26] reported that SP-A protein detection in tumour tissue sections was associated with lighter smoking history.

More evidence concerning surfactant protein A in pulmonary cancer has been provided by Mitsuhashi et al. [21], who, using human lung adenocarcinoma (PC14PE6, A549) lines transduced with the *SFTPA1* gene, demonstrated suppression of tumour progression in a murine model of subcutaneous xenografts or lung metastasis. They noticed a higher number of M1-type (M2-type unchanged) macrophages in tumours related to SP-A-synthesising cells, compared with a control. The number of natural killer cells was higher as well. It was suggested that SP-A induces polarisation of tumour-associated macrophages contributing to NK recruitment and activation and, finally, to the inhibition of tumour growth [21]. Recently, in silico analysis revealed in turn that low *SFTPD* gene expression is correlated with alectinib resistance and may predict poor prognosis in patients suffering from lung adenocarcinoma. An association of low SP-D expression and drug resistance was further confirmed experimentally (RT-PCR) with the use of the adenocarcinoma H3122 cell line and its alectinib-resistant counterpart H3122R [55]. This is in agreement with an earlier report published by Mangogna et al. [56], who, using the Oncomine^TM^ platform, noted a lower *SFTPD* mRNA level in lung cancers compared with normal tissue. They moreover suggested that SP-D expression in adenocarcinoma and squamous-cell carcinoma tissues may correlate with overall survival rate and therefore may be associated with a favourable prognosis. It was however in general lower in cancerous compared with normal tissue, as demonstrated with the use of immunohistochemistry [56]. Table 1 summarises the major mechanisms involved in anti-cancer potency of pulmonary surfactant collectins.

### 3.2. Cancers of Other Organs

Many fewer data have been collected and published with regard to the role of pulmonary collectins in primary tumours localised outside the respiratory system. Most of those reports have been focused on reproductive or digestive systems.

According to recent statistics, provided by the Global Cancer Observatory (GLOBOCAN), prostate cancer is the fourth most common cancer (nearly 1.5 million new cases in 2022 worldwide) and the eighth most deadly one (almost 0.4 million deaths) (7.3% and 4.1%, respectively) [19]. Most are adenocarcinomas. Expression of both SP-A and SP-D in the human prostate was noted in several studies [65,66,67,68,69,70]. Kankavi et al. [71], using immunohistochemistry, found lower SP-A and SP-D staining in prostate adenocarcinoma tissue sections compared with non-malignant tissue. They moreover reported lower expression of both collectins in relation to higher Gleason score, tumour volume and patient age. Very weak or no staining corresponded to a Gleason score ≥7. Those findings suggested that pulmonary collectins may be considered as disease markers [71]. Later, Thakur et al. [57] found lower *SFTPD* mRNA and SP-D protein in cells of the androgen-dependent prostate cancer LNCaP line compared not only with primary prostate epithelial cells but also with androgen-independent lines (PC3 and DU145). Furthermore, with the help of a TRAMP (transgenic adenocarcinoma of mouse prostate) model, increased degradation of SP-D (suspectedly via serine proteases synthesised by granulocytes and polymorphonuclear myeloid-derived suppressor cells) in advanced disease stage was evidenced [58]. Interestingly, a recombinant fragment of human SP-D (rfhSP-D, a trimer of chains consisting of CRD, neck domain and eight Gly-X-Y triplets) induced apoptosis of cells of both androgen-dependent (LNCaP, p53-wild-type) and -independent (PC3, p53-mutated) lines, explants and primary tumour cells from metastatic patients [57], as well as TRAMP explants [58], suggesting the therapeutic potential of that collectin. A 78 kDa glucose-regulated protein (GRP78) was identified as a ligand for the SP-D lectin domain in androgen-independent metastatic prostate cancer cells (PC3 line), based on interactome and docking analysis. Furthermore, recombinant GRP78 was shown to inhibit binding of CRD-specific anti-SP-D antibodies to the recombinant (complete molecule) collectin [63].

Testicular cancer is less common than prostate cancer (although >72,000 new cases and approx. 9000 deaths were noted in 2022) [19], but it is the commonest tumour among men aged <40 years [70]. SP-A and SP-D (as well as SP-B and SP-C) expression was demonstrated in normal, peritumoral and tumoral testes. Although there was no greater difference in the case of mRNA related to pulmonary collectins, the protein levels (especially SP-D in samples from seminoma patients) were generally lower in cancer cases [70].

The second most common malignancy worldwide (and the commonest in women but rare in men) is breast cancer (>2.3 million females were newly diagnosed and nearly 665,000 died in 2022) [19]. Its most common types include ductal breast carcinoma (in situ or invasive) and lobular carcinoma (in situ or invasive). Based on bioinformatics analysis (Oncomine^TM^ platform), Mangogna et al. [56] demonstrated lower *SFTPD* gene-related mRNA expression in invasive ductal breast carcinoma, male breast carcinoma and breast phyllodes tumours than in normal breast tissue samples. However, high SP-D expression was associated with shorter overall survival in patients with Luminal-A grade-1 and -2 breast cancers [56]. Like prostate cancer, SP-D may act pro-apoptotically on some breast tumour cells. The aforementioned rfhSP-D was demonstrated to induce cell death in HER2-receptor-over-expressing SKBR3, triple-positive (HER2, oestrogen and progesterone) BT474 lines. That activity was however inhibited efficiently in the presence of hyaluronic acid (promoting breast cancer progression and invasion when increased in the tumour microenvironment). Moreover, no pro-apoptotic effect was noted in the case of triple-negative cells of the BT20 line [59].

Ovarian cancer (OC), although much less common, is one of the deadliest tumours among women (>324,000 new cases and almost 207,000 deaths in 2022) [19]. The most frequent serous ovarian adenocarcinoma belongs to the epithelial OC type. The presence of SP-D in human ovaries was originally reported by Oberley et al. [72].

In the above-cited paper, a higher *SFTPD* mRNA expression in ovarian cancer (serous adenocarcinoma) compared with normal ovarian tissue was reported [56]. They confirmed that message experimentally, at both mRNA and protein levels. Additionally, more SP-D-expressing cells were found in the tumour microenvironment than in normal tissue [56]. Later, also based on Oncomine^TM^, Kumar et al. [73] described SP-D-specific mRNA expressed not only in serous ovarian adenocarcinoma but also in mucinous, clear cell and endometrioid-type tissues, independently of grade or stage. Although, generally, there was no significant difference in the expression levels in tumour tissue compared with normal tissue, high *SFTPD* mRNA in cases predicted shorter overall and progression-free survival. Immunohistochemistry confirmed in silico data: the SP-D protein was detected in serous papillary, mucinous and endometrioid cystadenocarcinoma samples. There were no significant differences depending on the tumour grade (1–3); however, high SP-D expression was over-represented among stage II compared with stage I samples. Moreover, low SP-D predicted better prognosis in patients at stages I–II but not III–IV. The protein appeared to be detectable not only in tissue sections (immunohistochemistry) but also in circulating tumour cells [73]. Importantly, like the TRAMP model, a recombinant fragment of human SP-D induced cancerous cell death, as evidenced with the use of the SKOV-3 line. It furthermore impaired SKOV-3 cells’ migratory capacity and affected mTOR (mechanistic target of rapamycin) signalling (via down-regulation of Rictor and Raptor, constituents of mTORC1/mTORC2 complexes), associated with ovarian cancer progression [73]. Those data were further confirmed and extended with the use of rfhSP-D immobilised on carbon nanotubes (CNTs) which inhibited Rictor and Raptor expression and induced synthesis of proinflammatory cytokines (IL-1β, TNF-α, TGF-β and GM-CSF) in SKOV-3 culture [74]. Altogether, data presented by Kumar et al. [73] and Alshaya et al. [74] again suggested the usability of SP-D as a disease marker and/or its recombinant fragment as a therapeutic agent.

Several associations of SP-D with gastrointestinal malignancies have been published. Colorectal cancer is the third most common and the second most lethal human cancer (over 1.9 million newly diagnosed cases and over 0.9 million deaths worldwide in 2022) [19]. Its most frequent type is adenocarcinoma. Although, to our knowledge, no papers concerning clinical material are available to date, some interesting data have been provided from a murine model. Tajima et al. [75], using the CMT93 (mouse rectal carcinoma) cell line, demonstrated significantly higher susceptibility of SP-D-knockout mice to developing pulmonary metastases compared to wild-type animals. Furthermore, the ability of murine recombinant SP-D to suppress the proliferation, migration and invasiveness of CMT93 cells was demonstrated [75].

Gastric cancer (almost 970,000 new cases (mostly adenocarcinomas) and almost 660,000 fatal outcomes in 2022) is the fifth most common and deadly type of tumour [19]. Mangogna et al. [56], based on bioinformatics analysis, reported lower *SFTPD* mRNA expression in cancerous gastric mucosa (intestinal, diffuse and mixed-type adenocarcinomas) compared with healthy tissue. A high level of SP-D expression predicted shorter overall survival in HER2-negative patients suffering from intestinal-type adenocarcinoma without distant metastases [56].

Less common (>510,000 new cases in 2022 worldwide, twelfth in frequency) but relatively more fatal (nearly 470,000 deaths, sixth most deadly) is pancreatic cancer [19]. Depending on the type of cells undergoing transformation, exocrine (including most common adenocarcinomas) and endocrine tumours may be diagnosed. As in the case of several other diseases discussed above, some promising data have been published concerning the therapeutic potential of a SP-D recombinant fragment. Kaur et al. [60] demonstrated a pro-apoptotic activity of rfhSP-D against pancreatic cancer cells, independently of their p53 status—namely, that product induced cell death via the Fas-mediated pathway in Panc-1, MiaPaCa-2 (both aggressive, p53-mutated) as well as Capan-2 (non-aggressive, p53-wild-type) cells. Importantly, a stronger effect was observed in the case of aggressive cells. The same authors provided evidence for the ability of rfhSP-D to suppress epithelial-to-mesenchymal transition (EMT) of the mentioned cells. Down-regulation of TGF-β as well as mesenchymal markers (vimentin, ZEB1 and Snail) was demonstrated [64].

Regarding cancers outside the reproductive or digestive systems, higher SP-A (as well as SP-C) concentrations were found in BALF samples from children suffering from various types of haematologic malignancies (mostly diagnosed with acute myeloid leukaemia or acute lymphocytic leukaemia) and immunosuppression during therapy, compared with controls. The difference appeared particularly significant in the case of patients with proven presence of pathogens in their material. Therefore, up-regulation of SP-A may be associated with both response to cancer and to infection. No differences in SP-D levels were found [76]. Later, Mahajan et al. [61,62] reported induction of p53-dependent apoptosis of AML.14.3D10 cells of eosinophilic leukaemia line by SP-D (derived from amniotic fluid) and its recombinant fragment.

As mentioned, Table 1 summarises the major mechanisms of SP-A and SP-D anti-cancer activity.

## 4. Concluding Remarks

Surfactant collectins as components of pulmonary surfactant and factors of innate immunity are important for maintaining homeostasis. Based on the literature cited here, it may however be stated that even though they generally act as Dr Jekyll (having anti-apoptotic activity and protecting from primary cancer/EMT/metastasis), under certain circumstances, they may play the role of Mr Hyde, when their high expression is associated with disease itself or predicts poor prognosis. Their widely understood relationships with cancer include a contribution to elimination of some carcinogenic pathogens (not discussed in this review). Finally, in some cancers, they may be considered promising diagnostic/prognostic markers, therapeutic agents or therapeutic targets. The recombinant SP-D fragment (rfhSP-D) has been shown to induce apoptosis of a variety of tumour cells and seems especially hopeful for the development of future therapies.

## Figures and Tables

**Table 1:** The major mechanisms of anti-tumour activity of pulmonary surfactant collectins.

Mechanism/Protein	References
Contribution to elimination of carcinogenic pathogens and apoptotic cells (SP-A and SP-D, prevention of cancer in general)	[1,4,5]
Pro-apoptotic potency against some cancer cells (SP-D and rfhSP-D)	[56,57,58,59,60,61,62]
Induction of tumour-associated macrophage polarisation and contribution to the recruitment of NK cells (SP-A)	[21]
Interactions with EGFR (SP-D, via the lectin domain; SP-A, electrostatically)	[41,42]
Interaction with GRP-78 (SP-D, via the lectin domain)	[63]
Suppression of EMT (rfhSP-D)	[64]

## Data Availability

This is a review paper; no new data were created.
